# Latent TGFβ complexes are transglutaminase cross-linked to fibrillin to facilitate TGFβ activation

**DOI:** 10.1016/j.matbio.2022.01.005

**Published:** 2022-03

**Authors:** Michael P. Lockhart-Cairns, Stuart A. Cain, Rana Dajani, Ruth Steer, Jennifer Thomson, Yasmene F. Alanazi, Cay M. Kielty, Clair Baldock

**Affiliations:** a Wellcome Centre for Cell-Matrix Research, Division of Cell-Matrix Biology and Regenerative Medicine, School of Biological Sciences, Faculty of Biology, Medicine and Health, University of Manchester, Manchester Academic Health Science Centre, Manchester M13 9PT, UK; b Department of Biochemistry, Faculty of Science, University of Tabuk, Tabuk, Saudi Arabia

**Keywords:** TGFbeta activation, fibrillin, LTBP, transglutaminase-2

## Abstract

TGFβ superfamily members are potent growth factors in the extracellular matrix with essential roles in all aspects of cellular behaviour. Latent TGFβ binding proteins (LTBPs) are co-expressed with TGFβ, essential for correct folding and secretion of the growth factor, to form large latent complexes. These large latent complexes bind extracellular proteins such as fibrillin for sequestration of TGFβ in the matrix, essential for normal tissue function, and dysregulated TGFβ signalling is a hallmark of many fibrillinopathies. Transglutaminase-2 (TG2) cross-linking of LTBPs is known to play a role in TGFβ activation but the underlying molecular mechanisms are not resolved. Here we show that fibrillin is a matrix substrate for TG2 and that TG2 cross-linked complexes can be formed between fibrillin and LTBP-1 and -3, and their latent TGFβ complexes. The structure of the fibrillin-LTBP1 complex shows that the two elongated proteins interact in a perpendicular arrangement which would allow them to form distal interactions between the matrix and the cell surface. Formation of the cross-link with fibrillin does not change the interaction between latent TGFβ and integrin αVβ6 but does increase TGFβ activation in cell-based assays. The activating effect may be due to direction of the latent complexes to the cell surface by fibrillin, as competition with heparan sulphate can ameliorate the activating effect. Together, these data support that TGFβ activation can be enhanced by covalent tethering of LTBPs to the matrix via fibrillin.

## Highlights

• TGFβ is regulated via the formation latent complexes in the extracellular matrix.
• Fibrillin-1 is a substrate for transglutaminase-2 which forms a covalent link between fibrillin-1 and latent TGFβ complexes.
• Formation of the cross-link increases TGFβ activation in cell-based assays.
• Fibrillin may direct the latent TGFβ complexes to the cell surface for activation.
• The structure of the cross-linked LTBP1-fibrillin complex has a perpendicular arrangement to enable bridging long-range interactions between the matrix and cell surface.

## Introduction

Fibrillin-1 is a major component of elastic fibres where it confers long range extensibility and contributes to the elastic deformation of tissues [1]. Fibrillin-1 also plays a key role in tissue homeostasis through its interaction with growth factors such as transforming growth factor-β (TGFβ) [2]. Fibrillin binds to latent TGFβ-binding protein (LTBP) and through this interaction is involved in regulating extracellular TGFβ signalling [3]. Mutations in fibrillin-1 give rise to a spectrum of disorders associated with dysregulated TGFβ signalling termed fibrillinopathies, the most common of which is Marfan syndrome (MFS) [4].

The LTBPs are extracellular glycoproteins and members of the fibrillin superfamily. There are four LTBP isoforms, where LTBPs -1, -3 and -4 bind to proTGFβ (also termed the small latent complex (SLC)). LTBPs are co-expressed with the SLC and are covalently linked to the TGFβ prodomain, also known as the latency associated peptide (LAP). LTBPs are disulphide bonded to LAP via the TGFβ-binding domain (TB) at their C-termini producing the large latent TGFβ complex (LLC) [5,6]. TGFβ activation is thought to occur by mechanical disruption of LAP via integrin binding where linkage of LTBP1 to the matrix is required to resist the applied force [7], [8], [9]. Cell based experiments suggest that the traction force exerted by integrins on LAP is required for TGFβ activation, because activation is abolished by deletion of the disulphide link with LTBP1 or disruption of the interaction with the matrix, both of which are required for exerting force across LAP [10]. Therefore, the LTBP superfamily is essential not only for correct folding and secretion of TGFβ but is also required to provide resistant forces for mechanical activation of TGFβ.

The interaction between LTBPs and the matrix is thought to be mediated by fibrillin and fibronectin (FN). FN assembly is required for LTBP1 deposition and activation of TGFβ from the LLC [11,12], but the underlying mechanism is unclear. In a number of studies, direct interaction between LTBP1 and full-length FN has not been observed, but an LTBP1 N-terminal region (aa 21‐629) was shown to interact with FN [12]; the presence of the FN-EDA domain supports binding and increases incorporation of LTBP1 into the matrix [13], suggesting that conformation and presentation of FN-binding epitopes may be important. Initial matrix targeting of LTBP1 is thought to be dependent on FN, but subsequent transfer to fibrillin microfibrils has been indicated in *in vitro* cell-based studies [11,14,15].

The interaction between LTBP1 and fibrillin-1 is mediated by an N-terminal region on fibrillin which binds to a C-terminal region on LTBP1 [14,16]. In addition to regulating TGFβ, TGFβ-independent roles have been proposed for members of the LTBP family in fibrillin microfibril assembly and elastogenesis [17]. For example, LTBP2 does not interact with TGFβ [18], but has a role in stabilising fibrillin microfibril bundles in the eye [19]. Both LTBP2 and -4 have been implicated in the formation of elastic fibres. LTBP2 and -4 have some commonalities in their functional roles, as LTBP4 can compensate for LTBP2 in some tissues [20]. However, even though LTBP3 and -4 are both expressed in the lung, they can only partially compensate for each other in lung development suggesting that the LTBP family have only some over-lapping functions [21].

Tissue transglutaminase or TG2 is also required for the matrix incorporation of LTBP1 and for TGFβ activation from the LLC [22,23]. TG2 catalyses an enzymatic transamidation reaction that cross-links primary amines (e.g., lysine residues) to glutamine residues [24]. LTBP1 and the LLC are transglutaminase substrates forming LTBP1-mediated multimers [23,25]. The N-terminal region of LTBP1 is involved in TG2 cross-linking as N-terminal LTBP1 antibodies block TG2 dependent cross-linking of LLC or LTBP1 [23], and inhibition of TG2 reduces matrix incorporation of LTBP1 [26]. An N-terminal LTBP1 antibody reduced TGFβ signalling suggesting that incorporation of LTBP1 into the matrix is required for TGFβ activation [15,23,27]. Since FN binding and TG2 cross-linking require the N-terminal region of LTBP1, and as FN is a TG2 substrate, it has long been thought that LTBP1 and FN are involved in a TG2 mediated cross-link that is required for proper matrix incorporation of the LLC and TGFβ activation from the LLC. However, a C-terminal antibody raised to the region incorporating the fibrillin binding site also reduces TGFβ activation [23], suggesting that matrix incorporation of LLC and TGFβ activation involve interactions with both the LTBP1 N- and C-terminal regions. TG2 also has a significant role in elastic fibre assembly where TG2 cross-linking stabilises interactions between fibrillin molecules within the assembled fibrillin microfibril [28]. A covalent cross-link between tropoelastin and fibrillin is formed by TG2, which stabilises the flexibility of tropoelastin [29,30].

Here we analyse the potential for TG2 mediated cross-linking of LTBPs to both FN and fibrillin. We show that although cross-linking was not observed with FN, LTBP1 and fibrillin can be stabilised by a TG2 cross-link. This cross-link forms in the presence of the SLC and does not affect the interaction between the SLC and integrin αVβ6 but enhances Smad2-mediated TGFβ signalling.

## Results

### Transglutaminase-2 cross-links LTBPs 1 and 3 to fibrillin

**Fig. 1:**
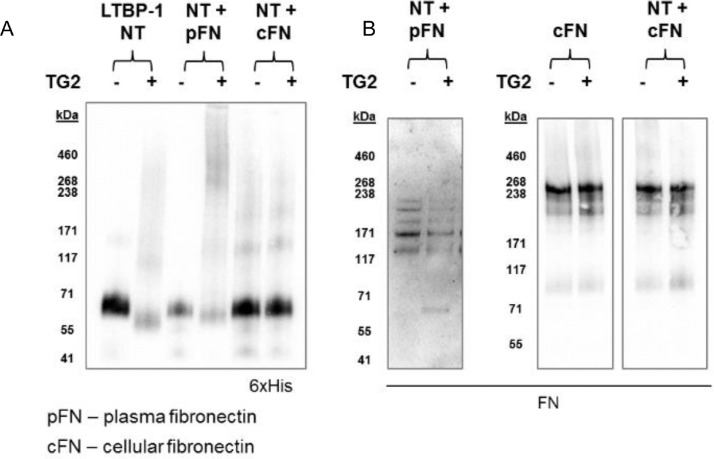
**A TG2 cross-link does not form between LTBP1 and fibronectin**. Western blotting of LTBP1 NT, plasma fibronectin (pFN) and cellular fibronectin (cFN) incubated with or without TG2. (A) The LTBP1 NT fragment was detected using a 6xHis antibody and (B) FN was detected using a monoclonal FN antibody. This assay was repeated at least three times (n=3).

TG2 cross-linking supports TGFβ activation where it is thought that TG2 cross-links LTBP1 to FN matrices [26]. Therefore, to investigate whether LTBP1 could be cross-linked to FN by TG2, cross-linking assays were performed using an N-terminal region of LTBP1 (LTBP1-NT) that contains the hinge region required for cross-linking, and either cellular or plasma FN. These cross-linking assays showed no higher molecular weight species were formed in samples containing LTBP1-NT and cellular FN, suggesting that they are not cross-linked by TG2 under the conditions utilised. Upon incubation of LTBP1-NT with plasma FN and TG2, western blotting detected some diffuse staining at larger than 250 kDa (Fig. 1). However, no clear bands were detected despite numerous repeats. The same result was observed with other constructs spanning the length of LTBP1 (SI Fig. 1) indicating a direct cross-link is not formed between LTBP1 and plasma or cellular FN in this assay.

**Fig. 2:**
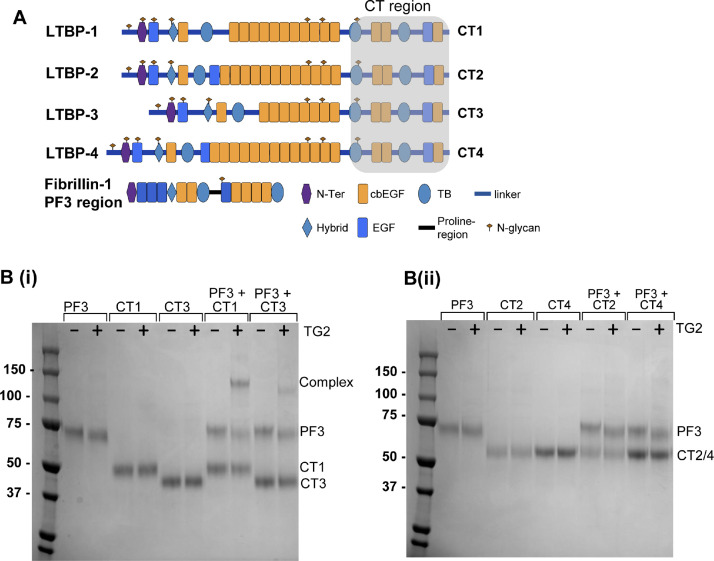
**LTBP1 and LTBP3 can be cross-linked to fibrillin by TG2**. (A) Schematic diagrams of the domain arrangements of LTBPs 1-4 and the C-terminal regions used in this study, referred to as CT1-4, respectively. These constructs along with the fibrillin1-PF3 region were expressed and purified from HEK293 cells. (B) SDS-PAGE gels, stained with Coomassie Blue, showing the fibrillin PF3 fragment and the C-terminal regions of LTBP1 (CT1), LTBP3 (CT3), LTBP2 (CT2) and LTBP4 (CT4) in the presence (+) and absence (-) of transglutaminase-2 (TG2). When incubated, complexes form between PF3-CT1 and PF3-CT3 (i) but not between PF3-CT2 and PF3-CT4 (ii). The two assays shown in (B) were performed at the same time, under the same conditions and with the same batch of TG2 but were run on separate gels.

Next, we tested whether LTBP1 and fibrillin could be cross-linked by TG2. Fibrillin is also a substrate for TG2, where TG2-cross-linking is important in microfibril and elastic fibre assembly and function [28,29,31]. To investigate whether LTBP1 could be cross-linked to fibrillin, constructs spanning their respective binding domains were expressed and purified from mammalian cells (Fig. 2A). The LTBP1 C-terminal region (LTBP1-CT or CT1) was incubated with an N-terminal region from fibrillin-1, referred to as PF3, in the presence or absence of TG2, to determine their capacity to be enzymatically cross-linked (Fig. 2B). CT1 showed no autologous cross-linking, but PF3 did form some higher molecular weight species in the presence of TG2 (SI Fig. 2). However, when CT1 and PF3 were mixed an intense higher band appeared, corresponding to the cross-linking of these proteins (Fig. 2B(i)). Western blotting showed that both proteins were present in this band (SI Fig. 2). Two over-lapping fibrillin constructs show that the cross-link is formed by the more N-terminal region of fibrillin (PF1), rather than the region that can cross-link to tropoelastin (PF2)[29, 30] (SI Fig. 2). Next, the other LTBP isoforms were also tested for their ability to form a cross-link with fibrillin. LTBP3-CT (CT3) also cross-linked to PF3 whereas LTBP-2 CT and -4 CT were unable to form cross-links with PF3. These data suggest that LTBP1 and LTBP3, the two LTBP isoforms predominantly involved in TGFβ regulation, can cross-link to fibrillin (Fig. 2B).

Interestingly, binding between fibrillin and LTBP3 was not detected in an earlier study [14], but the formation of a cross-link between these two proteins would suggest that binding can occur. Therefore, we probed for an interaction between the C-terminal region of LTBP3 and the PF3 and found that they do interact but with relatively low affinity (SI Fig. 3).

### Interactions and cross-links also form between latent TGFβ complexes and fibrillin

**Fig. 3:**
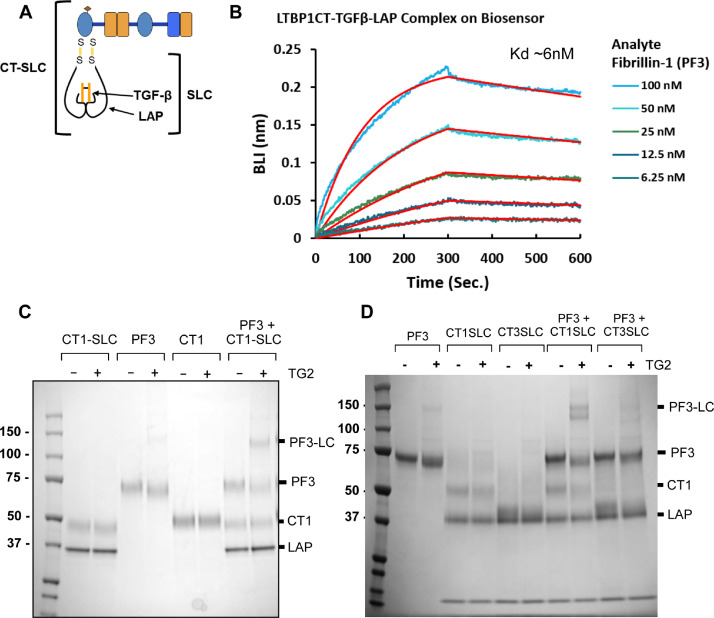
**The large latent TGFβ complex can be cross-linked to fibrillin to form larger complexes**. (A) Schematic diagram showing the CT1-SLC construct, where the LTBP1-CT region is co-expressed with SLC to form a truncated version of the large latent complex. (B) OctetRED Biolayer Interferometry (BLI) sensorgram showing the binding response detected between immobilized CT1-SLC and a range of concentrations (100-6.25 nM) of PF3. The binding affinity KD was determined by fitting to a 1:1 Langmuir binding model (red curves) and shows that fibrillin binds to LTBP1 with high affinity while part of the latent complex with TGFβ. Experiment was performed in duplicate and representative results are shown. (C) Reduced SDS-PAGE gel, stained with Coomassie Blue, showing the CT1-SLC, PF3, CT1 in the presence and absence of TG2. When PF3 and CT1-SLC are incubated in the presence of TG2, a larger species is formed indicating that cross-linking still occurs between PF3 and LTBP1 in the presence of latent TGFβ. (D) Reduced SDS-PAGE gel, stained with Coomassie Blue, showing the CT1-SLC or CT3-SLC with PF3 in the presence and absence of TG2 showing that cross-linking can also occur between PF3 and LTBP3 in the presence of latent TGFβ.

To assess whether the formation of the LLC changed the binding affinity between LTBP1 and PF3, binding assays were performed. A truncated version of the LLC, containing the LTBP1-CT region disulphide bonded to the TGFβ1-SLC was expressed and is referred to as CT1-SLC (Fig. 3A). This construct was used in preference to the LLC due to higher expression levels. A previous study using surface plasmon resonance demonstrated that the fibrillin-1 N-terminal region binds strongly to the immobilized LTBP1 C-terminal region with low nM affinity [16]. An OctetRED Biolayer Interferometry (BLI) assay probed the interaction between immobilised CT1-SLC and fibrillin. Our sensorgrams showed tight binding between immobilized CT1-SLC to a range of concentrations of PF3, indicating that the interaction of SLC with LTBP1 does not affect the fibrillin interaction. The dissociation constant K_d_ was ∼6 nM when fitted to a Langmuir 1:1 binding model indicating a high affinity interaction (Fig. 3B). Since a cross-link could be formed between fibrillin and LTBP1, we then wanted to test whether the cross-link still formed when LTBP1 is part of the LLC. TG2 cross-linking was performed using PF3 and CT1-SLC which showed the presence of a larger species indicative of a cross-link between LTBP1 and PF3 (Fig. 3C). Cross-linking was also performed with LTBP3-CT-SLC (CT3-SLC) which also formed a cross-link to fibrillin but less efficiently (Fig. 3D). These data show that the cross-links between LTBP1 CT or LTBP3 CT and fibrillin still formed in the presence of the SLC, indicating that LTBP1 or LTBP3 are still substrates for TG2 whilst part of the LLC, and that the presence of the SLC did not inhibit TG2 cross-linking to fibrillin.

### LTBP1 self-association competes with fibrillin interaction

**Fig. 4:**
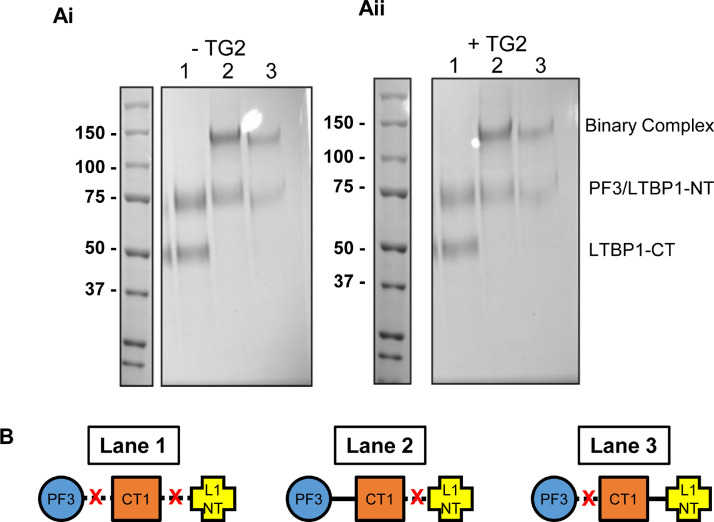
**LTBP1 self-association competes with fibrillin interaction**. (A) SDS-PAGE gels, stained with Coomassie Blue, assessing TG2 cross-linking of PF3, CT1 or the N-terminal region of LTBP1 (L1-NT) as either individual components or binary complexes. PF3, CT1 and L1-NT were mixed in the absence (i) or presence (ii) of TG2 (lane 1). In lane 2, PF3-CT1 binary complexes were mixed with L1-NT in the absence (i) or presence (ii) of TG2. Whereas in lane 3, L1NT-CT1 binary complexes were mixed with PF3 in the absence (i) or presence (ii) of TG2. In all cases no additional species, larger than the binary complexes, were formed as illustrated in (B), indicating that LTBP1 can either be involved in fibrillin cross-linking or LTBP1 self-association. Furthermore, no binary complexes were formed when all three components were mixed simultaneously (lane 1), indicating that interactions between the CT1 region and PF3 or L1-NT prevents TG2 cross-linking.

LTBP1 also forms intermolecular cross-links in the presence of TG2 via N- to N-terminal and C- to N-terminal interactions [25], so we asked whether ternary complexes incorporating fibrillin could be formed with these multimers. To determine whether LTBP1-LTBP1 complexes can be further cross-linked to fibrillin, or if the fibrillin-LTBP1 complex can associate with other LTBP1 molecules, cross-linking experiments were performed. LTBP1-CT, LTBP1-NT and PF3 were mixed in equimolar amounts, with and without TG2 (Fig. 4A, lane 1). Unexpectedly, when all three components are added together, SDS-PAGE analysis indicated that they do not form any higher order complexes, so binary LTBP1-LTBP1 or LTBP1-fibrillin complexes were not formed (Fig. 4A(ii) and Fig. 4B, lane 1). Next, we pre-formed binary complexes of either CT1-PF3, or CT1-LTBP1-NT and mixed these with equimolar amounts of either LTBP1-NT or PF3 respectively (Fig. 4A, lanes 2 and 3). When these complexes were preformed and mixed with the additional component no higher order species were observed (Fig. 4A(ii) and B, lanes 2 and 3). These data indicate that when LTBP1 CT is cross-linked to fibrillin it cannot be further cross-linked to LTBP1 NT, and conversely when LTBP1 is cross-linked to another LTBP1 molecule it cannot cross-link to fibrillin. These data suggest discrete roles for LTBP1 in either interacting with fibrillin or self-association and suggests that either the components may compete for interactions or that the three components interact in such a way to make TG2 cross-linking unfavourable when in a complex.

### Structure of the LTBP1 - fibrillin cross-linked complex

**Fig. 5:**
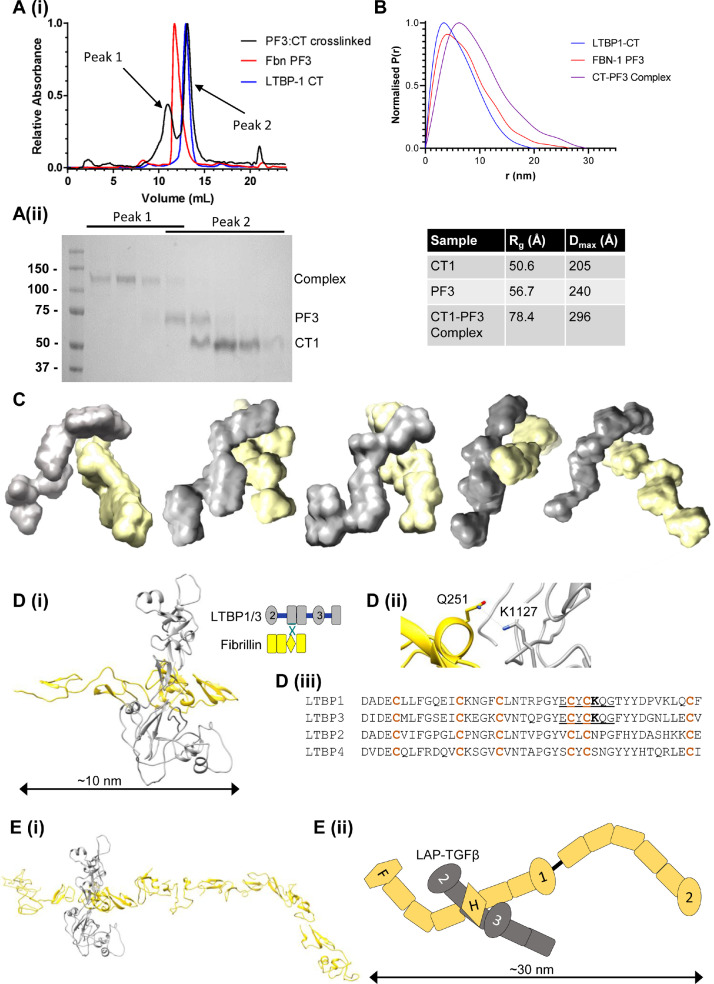
**Structure of the LTBP1 - fibrillin cross-linked complex**. (Ai) The size exclusion chromatography (SEC) profile of PF3 (red) and CT1 (blue) alone and after TG2 treatment (black). After TG2 treatment, a larger species is formed (peak 1). When run on SDS-PAGE (ii), the SEC elutions following TG2 treatment correspond to the CT1-PF3 complex (peak 1) and the two individual components (peak 2). (B) SEC-small-angle X-ray scattering data were collected for CT1, PF3 and the CT1-PF3 complex. The indirect Fourier Transform from the pair distance distribution function shows that PF3 and CT1 are similar in size but the complex is larger. The radius of gyration (Rg) and maximum dimension (Dmax) determined from the experimental SAXS data are indicated. (C) Multiphase modelling shows a perpendicular interface between the two proteins, CT1 (grey) and PF3 (yellow), within the complex. Five representative models are shown. (D(i)) The highest ranked AlphaFold model of the complex between LTBP1 (residues 1021 - 1331) in grey and fibrillin (residues 115 – 287) in yellow showing a perpendicular arrangement. (ii) A close-up view of the proposed region of TG2 crosslinking where K1127 in domain cbEGF13 of LTBP1 is in proximity of Q251 in the hybrid 1 domain of fibrillin. (iii) Sequence alignment shows that this lysine residue is conserved between LTBP1 and LTBP3 but not in LTBP2 and LTBP4. (E(i)) Model of the CT1 region (grey) and fibrillin PF3 region (yellow) based on published SAXS models of CT1 and the fibrillin PF2 region [25,29] and NMR structures of the fibrillin N-terminal region [32,35]. The modelling was informed by the AlphaFold model of the fibrillin-LTBP1 interface. (ii) Cartoon representation of the domain arrangement within the CT1-PF3 complex. H = hybrid domain; F = Fibrillin unique N-terminal region. The TB domains in fibrillin and LTBP1 are numbered.

To analyse the structure of the CT1-PF3 complex, the TG2 cross-linked complex was separated by size exclusion chromatography (SEC) (Fig. 5A). The complex and CT1 and PF3 alone were analysed by inline SEC-small-angle X-ray scattering (SAXS). SEC-SAXS data were collected at the European Synchrotron Radiation Facility and the data analysed using Guinier plots and the pair distance distribution function (Fig. 5 and SI Fig. 4). The maximum dimension (Dmax) from the pair distance distribution function shows that PF3 and CT1 alone are similar in size, 240 Å and 205 Å, respectively whereas the complex is larger (296 Å) (Fig. 5B). Similarly, the radius of gyration (Rg) for the complex (78.4 Å) is larger than PF3 (56.7 Å) and CT1 (50.6 Å). Ab initio modelling revealed an L-shaped structure for PF3 and a shorter elongated structure for CT1, consistent with previously published data [25]. Multiphase modelling was performed to analyse the structure of the complex, which shows both elongated proteins interacting in a perpendicular cross-shaped arrangement (Fig. 5C). This interface between CT1 and PF3 resembles the model proposed by Robertson and colleagues [32] which models the interaction between the fibrillin hybrid1 domain and LTBP1 TB3 domain. Sequence analysis was performed to find conserved glutamine or lysine residues in the TB3 domain of LTBP1 and -3, to try and identify the residue involved in TG2 cross-linking to fibrillin (SI Fig. 5). This analysis highlighted residue Q1561 which is conserved between LTBP1-3 and also showed a large chemical shift upon binding to fibrillin [32], suggesting it is located in the fibrillin binding interface. This residue was mutated to an asparagine, to determine the effect on cross-linking. However, this mutation did not prevent cross-linking to fibrillin, suggesting that if it is in the fibrillin binding interface it is not the glutamine residue involved in cross-link formation (SI Fig. 5).

We then used Deepmind AlphaFold-Multimer [33,34] to predict the structures of fibrillin-LTBP1 and fibrillin-LTBP3 complexes. The input domains from fibrillin were EGF2-3-hybrid1-cbEGF1 which have been shown to contain the LTBP1 binding epitope [16], and a 5 domain region TB2-EGF1 from LTBP1 or LTBP3 where the fibrillin binding site is thought to reside in TB3. The resulting models of the complex (Fig. 5D) show a perpendicular arrangement of domains which is consistent with the SAXS model. Close inspection of the interface between LTBP1 and fibrillin shows that the cbEGF13 domain on LTBP1 is also involved in the interaction, where a conserved lysine residue (K1127) is in close proximity to a glutamine residue (Q251) in the hybrid domain of fibrillin. The lysine residue is conserved in LTBP3 (K1072) but is not conserved in LTBP2 or LTBP4 which suggests why TG2 cross-linking does not occur for these isoforms (Fig. 5D (iii)). We extended the AlphaFold model to encompass the entire CT1 region and fibrillin PF3 region using our published SAXS models [25,29] and the NMR structure of the fibrillin N-terminal region [35]. This CT1-PF3 model resembles the cross-shaped structure predicted by multiphase modelling (Fig. 5E). The model for CT1 has similar dimensions to the SAXS model, whereas the model for the PF3 region is longer than that predicted by multiphase modelling, which is presumably due to the flexibility of this region, but consistent with the Dmax for the complex. The perpendicular arrangement at the interface of these proteins could allow them to participate in distal interactions bridging interaction partners. For instance, the distance between the N-terminal HS-binding domain cbEGF1 of fibrillin [36] and the TB2 domain of LTBP1 which is covalently linked to LAP is ∼6 nm.

### Fibrillin cross-linking enhances TGFβ signalling via Smad2 by promoting LLC access to the cell surface

**Fig. 6:**
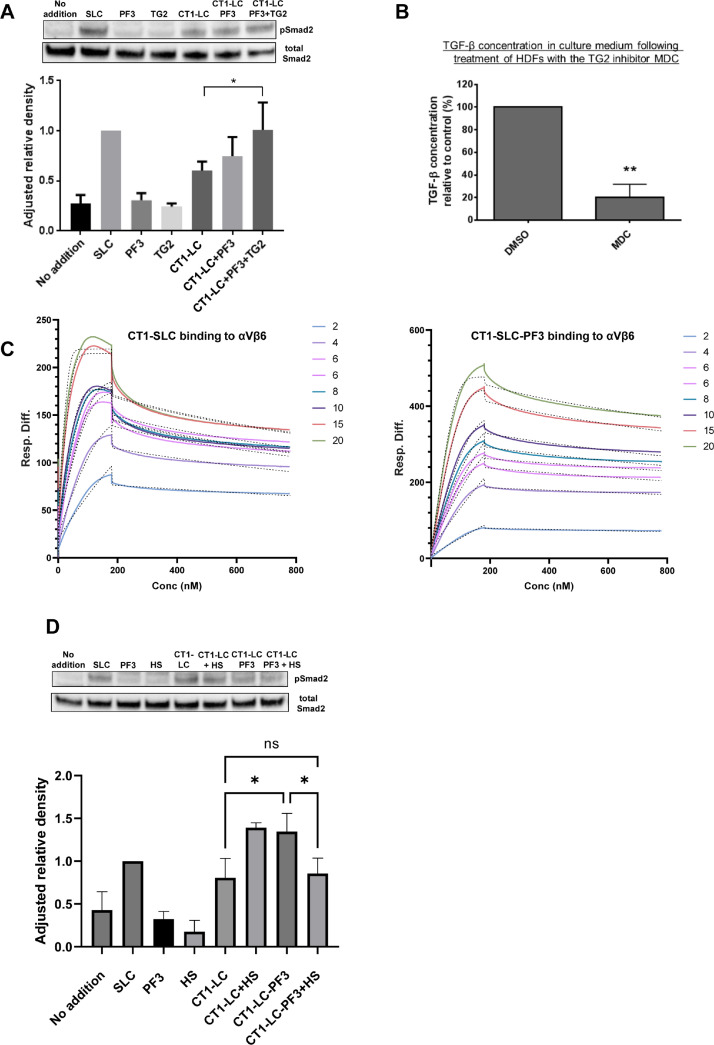
**Cross-linking the latent TGFβ complex to fibrillin directs TGFβ to the cell surface for activation**. (A) Western blotting to assess Smad2 phosphorylation compared to total Smad2 protein levels in HDFs treated with PF3, CT1-SLC, CT1-SLC plus PF3 or CT1-SLC-PF3 cross-linked complex. For each experiment, pSmad2 levels were analysed by densitometry and normalised to total Smad2 protein levels, with mean values for each treatment expressed as a proportion of SLC ± SEM. (B) HDFs were cultured for 5 days with DMSO (Control) or 100 μM MDC in DMSO. Media were analysed by ELISA for active TGFβ and shown as a percentage relative to the control. Results shown are representative of three independent experiments (n=3). ** indicates P < 0.01 from an unpaired T-test. (C) Surface plasmon resonance curves showing the binding response detected between either immobilised CT1-SLC or CT1-SLC-PF3 and integrin αVβ6. Proteins were immobilised via the Twin-Strep tag onto a Strep-Tactin XT surface and a range of concentrations (20-2 nM) of integrin αVβ6 used as the analyte. The binding affinity KD was determined by fitting to a 1:1 Langmuir binding model (dotted lines). Experiments were performed in triplicate and representative results are shown. (D) Western blotting to assess Smad2 phosphorylation and total Smad2 protein levels in HDFs treated with CT1-SLC or CT1-SLC-PF3 complex with or without addition of 5-molar excess of HS. For each experiment, pSmad2 levels were analysed by densitometry and normalised to total Smad2 protein levels, with mean values for each treatment expressed as a proportion of SLC ± SEM. For (A) and (D) results shown are representative of three independent experiments (n=3). * indicates P < 0.05 as determined by 1-way ANOVA with Tukey's post-hoc test.

To determine the effect of cross-linking the latent TGFβ complex to fibrillin on TGFβ signalling, levels of Smad2 phosphorylation were assessed via western blotting after treatment of HDFs with CT1-SLC or CT1-SLC cross-linked to fibrillin-PF3. We noted that there was a reduction of pSmad2 levels from SLC to CT1-SLC. This finding is consistent with previous studies which show that the N-terminal hinge region of LTBP1, which is absent in CT1-SLC, is required for the activation of TGFβ from the LLC [10]. The impact of cross-linking CT1-SLC to fibrillin was assessed. When CT1-SLC was cross-linked to fibrillin this resulted in an increase in pSmad2 levels, indicative of increased TGFβ signalling, to levels comparable to the SLC (Fig. 6A). PF3 alone did not induce Smad2 phosphorylation above the level of the control indicating that recombinant fibrillin was not bound to TGFβ [37](Fig. 6A). The addition of fibrillin with the CT1-SLC, but without TG2 cross-linking, also did not result in an increase in TGFβ activity indicating that TG2 cross-linking is required. These data are consistent with previous findings showing that TG2 cross-linking increases TGFβ activation [22,23]. Conversely, when TG2 was inhibited in HDFs, using the inhibitor monodancylcadaverin (MDC), the level of active TGFβ in the media decreased (Fig. 6B).

Therefore, as TG2 cross-linking to fibrillin supports TGFβ activation we investigated whether cross-linking fibrillin to latent TGFβ enhances the interaction between LAP and integrin αVβ6 to potentially facilitate TGFβ activation. Integrin αVβ6 binds to an RGD sequence on LAP which provides a pulling force to partially unfold LAP allowing the active TGFβ growth factor to be liberated from the latent complex for signaling [7,8,10]. Surface plasmon resonance assays were performed to determine the binding kinetics between integrin αVβ6 and either CT1-SLC or CT1-SLC cross-linked to PF3. The interaction between integrin αVβ6 and CT1-SLC was high affinity which was not changed when CT1-SLC was cross-linked to fibrillin, suggesting that the formation of the cross-link between LTBP1 and fibrillin does not change the molecular interaction between LAP and integrin (Fig. 6C).

We then considered whether the function of TG2 cross-linking to fibrillin is instead to bring the latent complex to the cell surface for activation. To test this hypothesis, heparan sulphate (HS) was added to the pSmad assays. Fibrillin binds to the syndecan receptors on the cell surface via HS [38], [39], [40] so the addition of HS to these assays should compete with fibrillin for interaction at the cell surface. Indeed, addition of HS to the CT1-SLC- fibrillin cross-linked complex ameliorated the activating effect of fibrillin (Fig. 6D). In the presence of HS, the pSmad2 level was reduced to that of the CT1-SLC which might suggest that TG2 cross-linking to fibrillin targets the latent complex to the cell surface which supports TGFβ activation.

## Discussion

LTBP and fibrillin are essential organisers of elastic fibres, required for the formation and function of mammalian elastic tissues [1]. LTBPs regulate the bioavailability of TGFβ via the formation of latent complexes. These latent complexes bind to fibrillin and FN to sequester TGFβ growth factors within the matrix which is critical for tissue homeostasis and remodelling [3]. These matrix proteins are essential to regulate cell signalling, but an unresolved aspect of TGFβ signalling is the molecular events surrounding regulation of growth factor bioavailability and activation.

LTBP1 is known to be a TG2 substrate and has been shown to be covalently linked to extracellular matrix fibrils by TG2 which is required for TGFβ activation [23]. A cross-linking site was localised to the N-terminal region of LTBP1 [23] but the cross-linked matrix partner was not identified but thought to be FN. *In vitro* cross-linking assays with cellular and plasma FN using constructs spanning the length of LTBP1 indicated that a direct cross-link is not formed between LTBP1 and FN *in vitro*. This suggests that the cross-link may not be directly formed between LTBP1 and FN but mediated by another protein, or that cross-linking requires the cell surface environment. Previous studies have indicated that binding between LTBP1 and FN is not direct but mediated by HS [41,42] but others have shown interaction or enhanced binding with specific regions of FN [12,13]. Our assays used cellular FN which has the EDA domain, as well as plasma FN which does not. Furthermore, LTBP-FN binding can be enhanced by pre-incubation of LTBP1 with HS [13,41,42] where a conformational change upon HS binding may potentially make the FN binding site more available. So, it is possible that cross-linking may also require the presence of other proteoglycans or cell-surface syndecans to support the preferred conformation for these interactions.

Despite the role of TG2 in TGFβ activation and fibrillin microfibril assembly, TG2 -/- mice develop normally which suggests that they do not have elastic fibre defects or dysregulated TGFβ signalling [43,44]. It has been suggested that the calcium-dependent transamidation function could be compensated for by the other eight members of the transglutaminase family, some of which are found extracellularly [45]. In the TG2 -/- mice, deLaurenzi and Melino saw residual transglutaminase activity in liver and thymus, where TG1 (normally expressed in skin) was present. Their data suggests that TGase 1 activity or redundancy of other transglutaminases, can compensate for the lack of TG2 in the TG2 -/- mouse [43].

Given that LTBPs and fibrillin interact, and that fibrillin is also a transglutaminase substrate we tested whether a TG2-mediated cross-link could form between fibrillin and LTBPs. Our data show that fibrillin and LTBP-1 and -3 can participate in a TG2 mediated cross-link, between the C-terminal region of LTBP1 and N-terminal region of fibrillin. It was thought that the C-terminal region of LTBP3 did not interact with fibrillin [14] but in our assays binding did occur although the interaction was of relatively low affinity. A TG2 cross-link is not formed between LTBP2/4 C-terminal regions and fibrillin so appears only relevant to the LTBPs primarily involved in TGFβ binding, rather than the LTBPs with TGFβ-independent roles of assembly and stabilisation of microfibril bundles and elastic fibres [19,20]. Furthermore, cross-linking between LTBP1/3 and fibrillin occurs in the presence of the SLC, which did not alter the formation of LTBP-fibrillin complexes. We also analysed the interaction between fibrillin and LTBP1-SLC, to determine whether the presence of the SLC altered LTBP1-fibrillin binding, but the interaction between fibrillin and LTBP1-SLC was still high-affinity. These data suggest that cross-linking to fibrillin may be a way of stabilising latent TGFβ complexes in the matrix. Indeed, addition of a C-terminal LTBP1 antibody, which incorporates the fibrillin binding site, reduced TGFβ activation in cell-based assays [23] and also suggests that matrix incorporation of LLC and TGFβ activation involve interactions with both the LTBP1 amino- and carboxyl-terminal regions. Transglutaminase crosslinks occur extracellularly after deposition of LTBP and fibrillin microfibrils in the matrix so could be a mechanism to covalently link proteins that are expressed at different times, or fold independently. Whereas, disulphide crosslinks usually occur intracellularly in the ER, as is the case for the large latent TGFβ complex where disulphide bonding of proTGFβ to LTBP facilitates secretion of TGFβ. Formation of cross-linked LTBP-fibrillin complexes intracellularly may inhibit microfibril assembly but disulphide bonds may also form in the extracellular space or isomerisation of disulphide bonds may occur which could be another mechanism for the formation of extracellular complexes.

It has previously been shown that LTBP1 can cross-link to itself, forming higher-order multimers [23,25]. Therefore, competition assays were performed to probe the preference for LTBP1-LTBP1 versus LTBP1-fibrillin cross-linking and to determine if higher order LTBP1 multimers could be formed containing fibrillin. These assays indicated that LTBP1 can participate in either LTBP1-LTBP1 or LTBP1-fibrillin cross-links but not both. For instance, when LTBP1 is cross-linked to fibrillin it cannot be further cross-linked to LTBP1, and conversely when LTBP1 is cross-linked to another LTBP1 molecule it cannot be cross-linked to fibrillin. These data suggest discrete roles for LTBP1 in either interacting with fibrillin or self-association and suggests that the availability of binding sites at the C-terminus of LTBP1 may limit cross-linking to either the N-terminal region of LTBP1 or fibrillin. This may suggest that LTBP C-terminal interactions may be important in directing the specific role or function of LTBP1.

Force activation of TGFβ requires LTBP1 to facilitate interactions with the matrix for resistance to a pulling force [7,10]. Indeed, mutation of the LTBP1 interaction site on LAP or a lack of LTBP1 or -3 leads to decreased TGFβ activation indicating that formation of the LLC is essential for TGFβ regulation [46,47]. TGFβ is released from the LLC by integrin-mediated activation where interaction of LTBP with the matrix is required as part of a mechanosensing hub [7,12]. We tested what effect cross-linking of fibrillin to LTBP1-latent TGFβ complexes had on TGFβ activation or integrin binding. Cell-based TGFβ activity assays showed that TGFβ becomes progressively more latent (or less active) from the SLC to the CT1-SLC. This is consistent with the finding that the N-terminal matrix interaction region is required for activation of latent TGFβ [10]. Surprisingly, cross-linking the CT1-SLC to fibrillin did not increase the latency further but rather addition of cross-linked CT1-SLC-PF3 resulted in an increase in Smad2-dependent signalling suggesting that cross-linking to fibrillin is making the LLC more activatable. The TG2 cross-link was required as addition of fibrillin did not increase Smad2-dependent signalling either on its own or in the presence of CT1-SLC. We then asked why does cross-linking LTBP-SLC to fibrillin increase TGFβ activation, and whether cross-linking to fibrillin could be enhancing the integrin-LAP interaction. We therefore analysed the interaction between integrin αVβ6 and LAP as part of a LTBP1-latent complex, this was high affinity and did not change when LTBP1 was cross-linked to fibrillin as a ternary fibrillin-LTBP-SLC complex. Therefore, the increased activation of TGFβ when LTBP1-latent TGFβ is cross-linked to fibrillin is not due to a change in the affinity for integrin αVβ6.

We then asked why does fibrillin-LTBP1 cross-linking increase TGFβ signalling, if this is not by changing the interaction with integrin, could cross-linking to fibrillin be providing a matrix anchor for TGFβ activation and supporting an association with the cell-surface to bring LAP into proximity with integrin receptors. An earlier study showed that a construct containing only the N-terminal hinge region and LAP-binding domains of LTBP1 was sufficient for effective TGFβ activation by αVβ6 [10]. The hinge region was thought to provide matrix targeting properties via fibronectin and the LAP-binding domain the covalent linkage to the SLC. We wondered if fibrillin could be performing the matrix targeting function in the absence of the N-terminal hinge region, as fibrillin is thought to associate with the cell surface via HS-mediated interaction with syndecans [40]. To test whether we could perturb the interaction between fibrillin and the cell surface, HS was added to fibrillin-LTBP1-SLC to compete for fibrillin-binding to the cell surface. The addition of HS with fibrillin-LTBP1-SLC resulted in a reduction in TGFβ activation. Although in cell-based assays, we cannot rule out indirect involvement of other cell or matrix components, this reduction appeared to be mediated via fibrillin, as HS addition in absence of fibrillin increased TGFβ signalling, consistent with previous studies where HS inhibited incorporation of LTBP1 and increased active TGFβ [41].

**Fig. 7:**
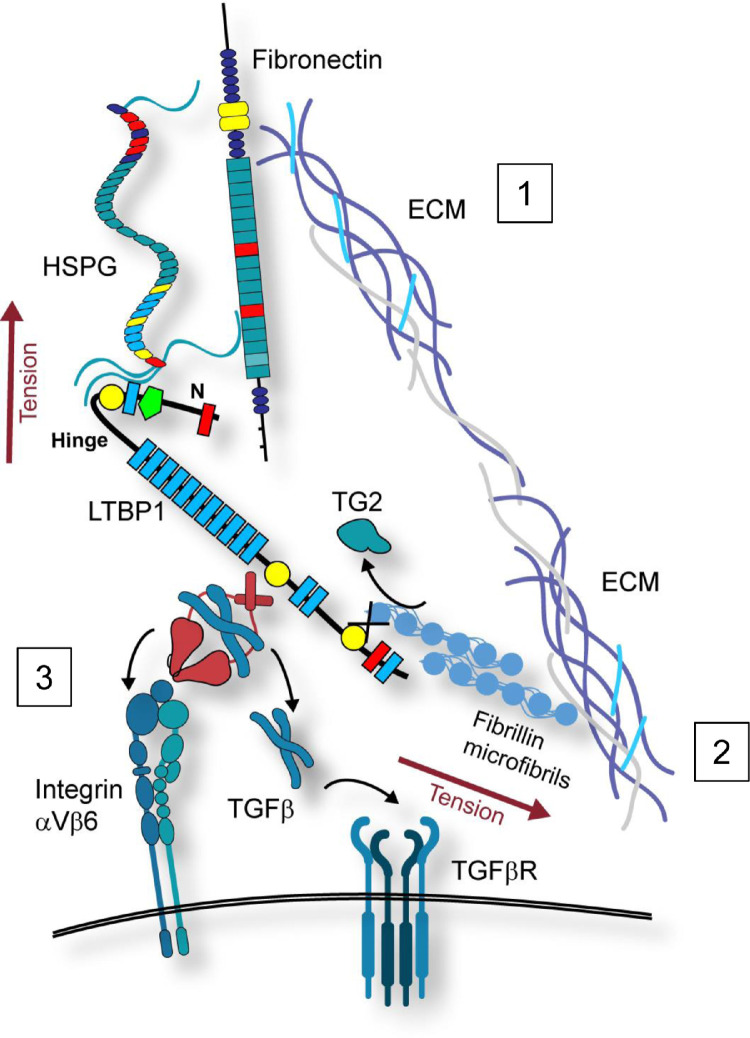
**Model showing TG2 cross-linking of latent TGFβ complexes to fibrillin or fibronectin-matrices facilitates TGFβ activation.** Cartoon showing how cross-linking the latent TGFβ complex to the matrix supports TGFβ activation. 1) Fibronectin and TG2 are required for activation of the LLC. The interaction with fibronectin which may be mediated through a heparan sulphate proteoglycan (HSPG), is thought to involve the LTBP1 hinge region as LLC constructs missing the hinge region are not activated and LTBP1 constructs lacking the N-terminal region are not incorporated into the matrix where it is thought that matrix targeting is needed for TGFβ activation. TG2 cross-linking increases TGFβ activation potentially by directing LLC complexes to the cell surface. This is thought to involve FN and HS interactions but fibronectin and LTBP1 are not directly cross-linked in vitro, suggesting an intermediary may be involved or conformational dependent interactions are required. 2) TG2 cross-linking of LTBP1 to fibrillin via the C-terminal region of LTBP1 provides another matrix anchor and supports TGFβ activation in the absence of the hinge region which explains why an antibody to the C-terminus of LTBP1 could block TGFβ activation. 3) Integrin binding to LAP (red) provides resistant forces between the cell surface receptor and matrix-tethered LLC which results in force activation of TGFβ leading to signalling via TGFβ receptors.

Our data suggest that as well as the N-terminal hinge region, the C-terminal region of LTBP1 may also be important in cell surface interactions and TGFβ activation, but mediated via fibrillin (Fig. 7). Indeed, from our SAXS data the size of the CT1-PF3 complex is ∼30 nm with the distance from the N-terminal HS-binding domain of fibrillin and the TB2 domain of LTBP1, which provides the covalent linked to LAP, is ∼6 nm. Integrin αVβ6 binds to LAP and HS-binding to fibrillin may be via syndecan receptors, and the length of the CT1-PF3 complex is within the size range to span these cell surface receptors [48]. Moreover, the flexibility of the C-terminal region of LTBP1 [25,49], may enable LTBP1 to make contacts with fibrillin microfibrils while additionally facilitating force-induced TGFβ activation via integrins. Our structure of the LTBP1-fibrillin complex (Fig. 5) would support this premise, where the perpendicular arrangement of the interface between these proteins could allow them to participate in distal interactions spanning the matrix to the cell surface. Modelling the complex with AlphaFold Multimer suggests that the interface between LTBP1 and fibrillin is more extensive than initially predicted, with domain cbEGF13 in LTBP1 also contributing to the interface. This finding is consistent with binding data that showed that constructs including this domain bound to fibrillin more tightly than constructs lacking this domain [14]. Together these data support a role for a TG2-mediated covalent interaction between LTBP1 and fibrillin in supporting TGFβ activation.

## Methods

### Protein expression and purification

The LTBP1 and fibrillin-1 constructs were expressed in HEK293-EBNA cells as previously described [25,36,50]. LTBP4-CT (aa1114-1557) was ligated into a modified version of the mammalian expression vector pCEP4 (Invitrogen), pCEP-Pu/AC7 that contains a C-terminal six-histidine residue tag (6x His-tag) and expressed in HEK293-EBNA cells. LTBP2-CT (NM_000428 aa1411-1821) and LTBP3-CT (NM_021070 aa917-1256) were cloned into a modified pCDH lentiviral vector (SBI, System Biosciences) with a C-terminal V5 and 6x His-tag. Virus was generated in 293T cells and then used to transduced HEK293-EBNA cells as previously described [51]. Protein fragments were harvested from serum free media and purified by nickel affinity chromatography using His Trap excel columns, (Cytiva), followed by size exclusion chromatography (SEC) on a Superdex 200 10/300 increase column in 10 mM Tris HCl pH 7.4, 150 mM NaCl, 2 mM CaCl_2_. For LTBP1-CT, the SEC buffer was 10 mM Tris, 150 mM NaCl pH7.4 with added equimolar amount CaCl_2_ to purified protein, sufficient to prevent degradation without causing aggregation. For PF3, the SEC buffer was 10 mM Tris, 500 mM NaCl, pH7.4.

LTBP1-CT Q156N mutant was cloned into a modified pCDH lentiviral vector with a C-terminal Twin-Strep-Tag and expressed in HEK293-EBNA cells. Harvested serum free media was purified using a Strep-Tactin XT 1ml column (IBA GmbH) and SEC. For the CT1-SLC construct, CT1 and SLC with N-terminal Flag Tag were both cloned into the dual expression vector pBUD4.1 (ThermoFisher) and co-expressed using HEK293T cells. The complex was purified from serum free media using Anti-Flag M2 Affinity resin (Merck) followed by SEC as described above. CT3-SLC was purified in a similar way by co-expressing LTBP3-CT with a C-terminal Twin-Strep-tag and Flag-SLC. The complex was purified from serum free media using a Strep-Tactin XT 1ml column (IBA GmbH) and SEC.

Human Integrin αV chain (NM_002210 aa31-992), was cloned with a C-terminal acid coiled coil and 6x His-tag into the lentiviral vector pCDH (with a tagBFP reporter). Human Integrin β6 chain (NM_000888 aa22-709) was cloned with a C terminal base coiled coil and MYC tag into another pCDH lentiviral vector with a tagGFP2 reporter. Viruses were generated for each chain and used to dual transduce HEK293-EBNA cells. Positive cells expressing both tagBFP and tagGFP2 were selected by FACS and then expanded. Integrin αVβ6 hetero-dimer formation was driven by interaction of the acid and base coiled coil and was purified using nickel affinity chromatography and SEC.

### TG2 cross-linking

A number of parameters were optimised for TG2 cross-linking assays including a concentration range of TG2 (0.1 – 0.3 ug), incubation time (3hrs and overnight) and temperature (4°C, 20 °C and 30 °C). The results shown for LTBP-fibrillin cross-linking represent the experiments optimised for the highest amount of cross-linked 1:1 complex where longer incubation times or higher amounts of enzyme resulted in higher molecular weight species. These conditions were also similar to those used in our study on TG2 cross-linking of fibrillin and tropoelastin [29]. Proteins were incubated with and without transglutaminase-2 (guinea pig liver, Merck) in a 1:5–1:10 mass ratio at room temperature for 3 hours. For cross-linking to LTBP, ∼1 µg of plasma FN (Millipore Ltd), cellular FN (Sigma-Aldrich) or PF3, were mixed in a 1:1 ratio with ∼1 µg LTBP1 with 0.2 µg of TG2 for analysis by SDS-PAGE. For purification and SEC-SAXS, a larger scale was used (∼0.5–1 mg of PF3 and CT1) and the resultant complex was separated using size exclusion chromatography on a Superose 6 column in 10 mM HEPES, 500 mM NaCl, pH 7.4 with equimolar amount of CaCl_2_ to purified protein.

### Small-Angle X-ray Scattering data collection and processing

Data were collected using inline SEC-SAXS on a Superdex 200 3.2/300 increase column (Cytivia) at the ESRF on Beamline BM29 and data collection and processing parameters are detailed in Supplementary Tables 1-3. The SEC column was run at a flow rate of 0.075 mL/min. Data for the run were collected at 1 second exposures and lasted the length of the SEC run. Images were reduced at the beamline and reduced 1D scattering curved were processed in the ATSAS suite. CHROMIXS [52] was used for automated sample and buffer selection and processed through PRIMUS [53,54]. PRIMUS was used to calculate the Guinier Rg and I(0) parameters, and plotting the Normalised Kratky. The PDDF Rg, I(0) and Dmax were then calculated, along with the mass of the samples. Multiphase ab initio modelling was conducted using MONSA [55] online. The parameters for each phase included the Rg and the mass of each component. The scattering curves used for each component and the complex were those obtained from PRIMUS after the PDDF was calculated. SAXS data and models are deposited in SASBDB under accession numbers SAS2942, SAS2943 & SAS2945 for CT1-PF3 complex, CT1 and PF3.

### Surface Plasmon resonance

All binding experiments were performed in 10 mM HEPES pH 7.4, 0.1 M NaCl and 0.05% surfactant P20 (designated HBS-P). LTBP-3-CT was immobilized by amine coupling onto CM5 sensor chips (Cytiva) in 50 mM sodium acetate buffer pH 5.5 at 30 μg/ml, giving typical immobilization of 3000 response units (RU). CT1-SLC-PF3 was immobilised via the Twin-Strep tag onto a Strep-Tactin XT immobilized surface. Strep-Tactin XT (IBA GmbH) was initially immobilised onto a CM5 sensor chip at a concentration of 5 ug/ml in 10mM sodium acetate buffer as specified by the manufacturer's instructions. For kinetic studies, protein fragments were injected at a flow rate of 30 μl/min and regeneration was performed by dual injections of 0.8 M NaCl. Binding was either calculated using 1:1 binding kinetics (Biacore T200 Evaluation Software V2.0) or independently using equilibrium analysis. Equilibrium response was plotted against concentration, and non-linear regression was used to calculate KD using the equation for one site binding.

### Bio-layer interferometry

Binding studies were performed on an OctetRED96 system (ForteBio) using biolayer interferometry (BLI). Streptavidin biosensors were hydrated in 10 mM HEPES, 150 NaCl, pH 7.4, 0.005% (v/v) Tween-20 for 10 minutes then loaded with biotinylated ligand at constant concentration. The loaded biosensors were incubated with serial dilutions of analyte in the same buffer used for hydration. The biosensors were regenerated using 10 mM glycine at pH 2.5. The background response was subtracted from all binding sensorgrams and all experiments performed in solid-black 96 well plates at 25^°^C with an agitation speed of 1000 rpm and repeated at least twice. The binding kinetics were analysed using Octet software version 7 (ForteBio). The goodness of binding data fitting was assessed by χ^2^ and R^2^ values.

### AlphaFold model generation

For the generation of LTBP and fibrillin models, AlphaFold multimer models were generated using a locally installed version of AlphaFold v2.1.1 [33,34] on the Computationally Shared Facility at University of Manchester. The installation included the full genetic databases for multiple sequence alignments. Sequences of LTBP-1 (Q14766-2:1021-1331), LTBP-3 (Q9NS15-2:917-1206) and fibrillin-1 (P35555-1:115-287) were used for modelling, around the proposed region of crosslinking. FASTA files containing complexes of LTBP-1 and fibrillin or LTBP-3 and fibrillin were prepared and submitted to the AlphaFold pipeline and ranked by the software.

### TGFβ assays

Western blotting was used to assess Smad2 phosphorylation compared to total Smad2 protein levels in HDFs. Proteins were added exogenously to HDFs (either TGFβ, SLC, 4 nM CT1-SLC, 4 nM CT1-SLC cross-linked to PF3, 80 nM PF3). Western blots were analysed by densitometry to quantify Smad2 phosphorylation with mean values for each treatment expressed as a proportion of pro-TGFβ controls. For each experiment, pSmad2 levels were normalised to total Smad2 protein levels. These assays were repeated with or without addition of 5-molar excess HS. Error bars are ± SEM from 3 independent experiments.

### Inhibition of transglutaminase-2 in HDFs

To investigate inhibition of TG2 in cell culture, neonatal HDFs were seeded at densities of 1.23 × 104 cells/cm2 (low), 2.46 × 104 cells/cm2 (medium) or 3.70 × 104 cells/cm2 (high) in 6 well plates. MDC (Sigma) was added at a concentration of 100 μM in 2 ml of medium (Nunes et al., 1997). An equal volume of DMSO was added to the control wells. Cells were cultured for 5 days before analysis by TGF-β1 Emax® ImmunoAssay system (Promega).

## Appendix Supplementary materials

Image, application 1
