# Supplementary material for: Latent TGFβ complexes are transglutaminase cross-linked to fibrillin to facilitate TGFβ activation
Source: Matrix Biol. 2022 Mar;107:24–39. doi: 10.1016/j.matbio.2022.01.005 (PMC8932414; doi:10.1016/j.matbio.2022.01.005)
Supplement: Supplementary file 1 [file mmc1.pdf]

Supplementary Figure 1

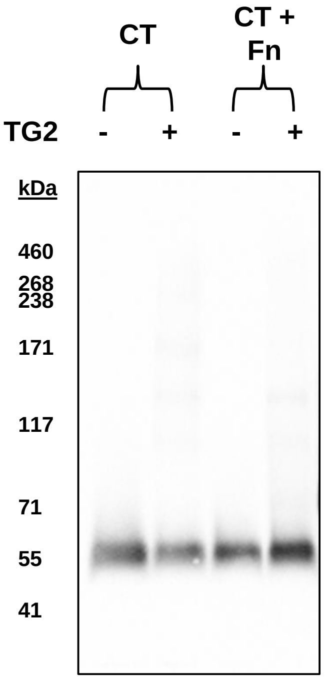

Western Blot analysis of LTBP1-CT and Fn incubated with or without TG2 for 3 hours. No higher molecular weight species are observed. This analysis was performed at least three times (n>3).

Supplementary Figure 2

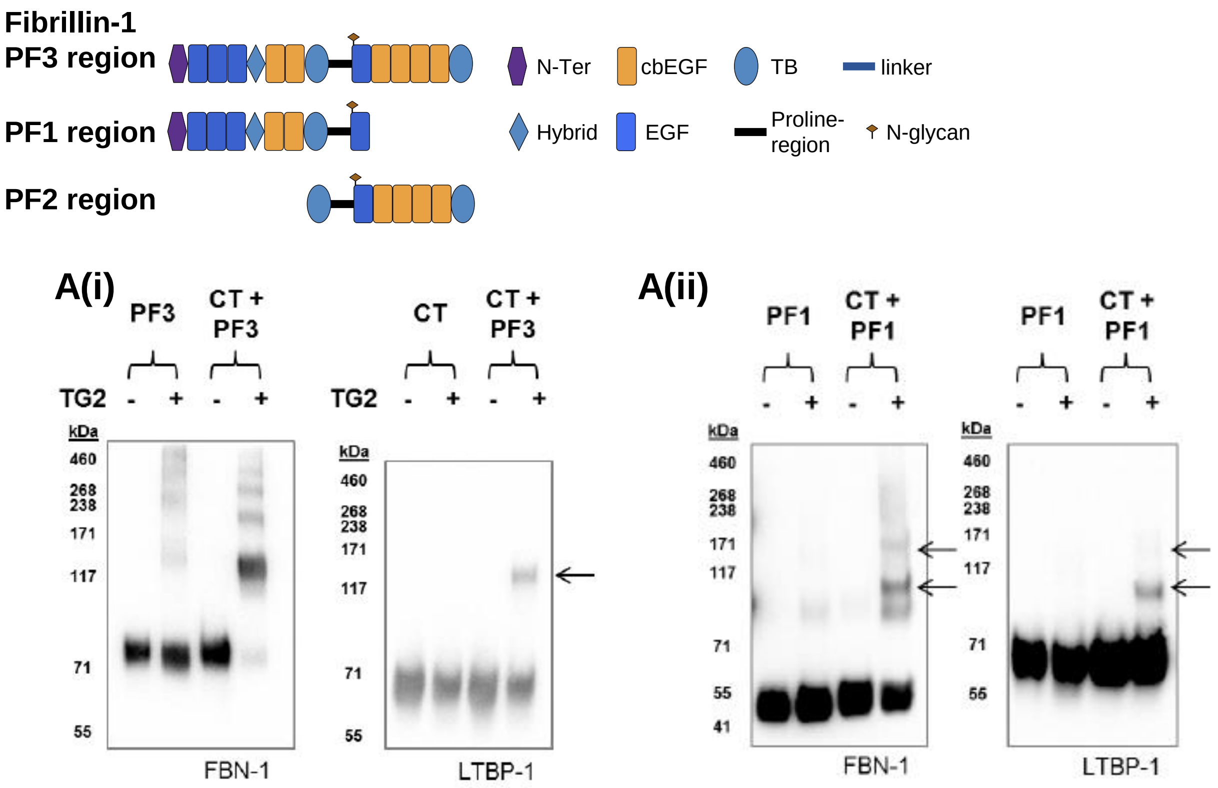

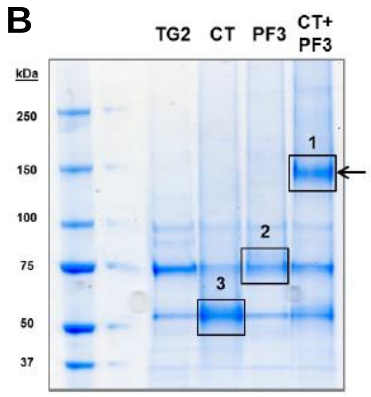

	Sample 1	Sample 2	Sample 3
Fibrillin-1 peptides	44	38	
LTBP-1 peptides	16		20

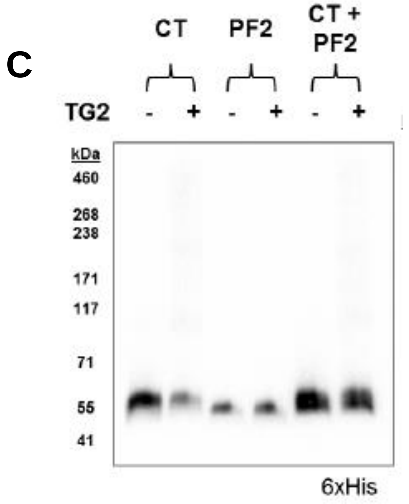

(A) Western Blot analysis of LTBP1-CT and fibrillin PF3 (i) or PF1 (ii) incubated with TG2 for 3 hours using fibrillin-1 and LTBP1 specific primary antibodies. In (i) the arrow shown on the LTBP-1 blot shows the presence of the LTBP1-CT:PF3 complex without formation of higher oligomers. The anti-fibrillin blot shows the formation of some higher order PF3 oligomers but an enrichment of the LTBP1-CT:PF3 complex. This analysis was performed at least three times (n>3). In (ii) the arrows depict higher molecular weight bands only detected when LTBP1-CT and PF1 were incubated with TG2. This analysis was performed twice (n=2).

(B) SDS-PAGE gel run under reducing conditions stained with Coomassie brilliant blue stain. The arrow depicts the 150 kDa band corresponding to the LTBP1-CT:PF3 complex. Black boxes show the bands excised for in gel digestion and mass spectrometry. LTBP1 and fibrillin peptides were both detected in the excised band from sample 1 as indicated in the table which shows the number of unique peptides identified. This analysis was performed once (n=1).

(C) Western blotting showing fibrillin-1 and LTBP-1 bands after incubation of LTBP1-CT and fibrillin PF2 with or without TG2. This analysis was performed three times (n=3).

Supplementary Figure 3

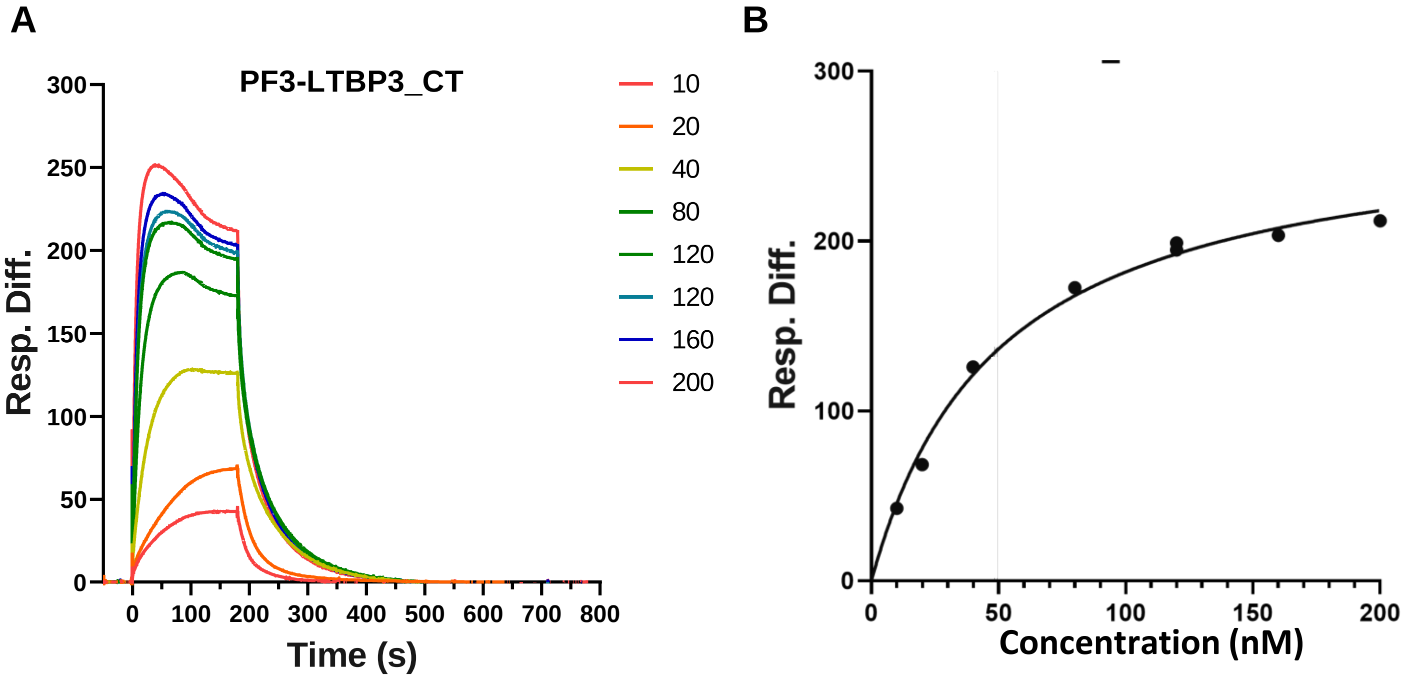

(A) Surface plasmon resonance curves showing the binding response detected between immobilised LTBP3-CT and a range of concentrations (200-10 nM) of fibrillin PF3 used as the analyte.

(B) The binding affinity KD was determined by equilibrium analysis where a KD of 49.7 +/- 5.5 nM was extrapolated. Experiments were performed in triplicate and representative results are shown.

Supplementary Figure 4

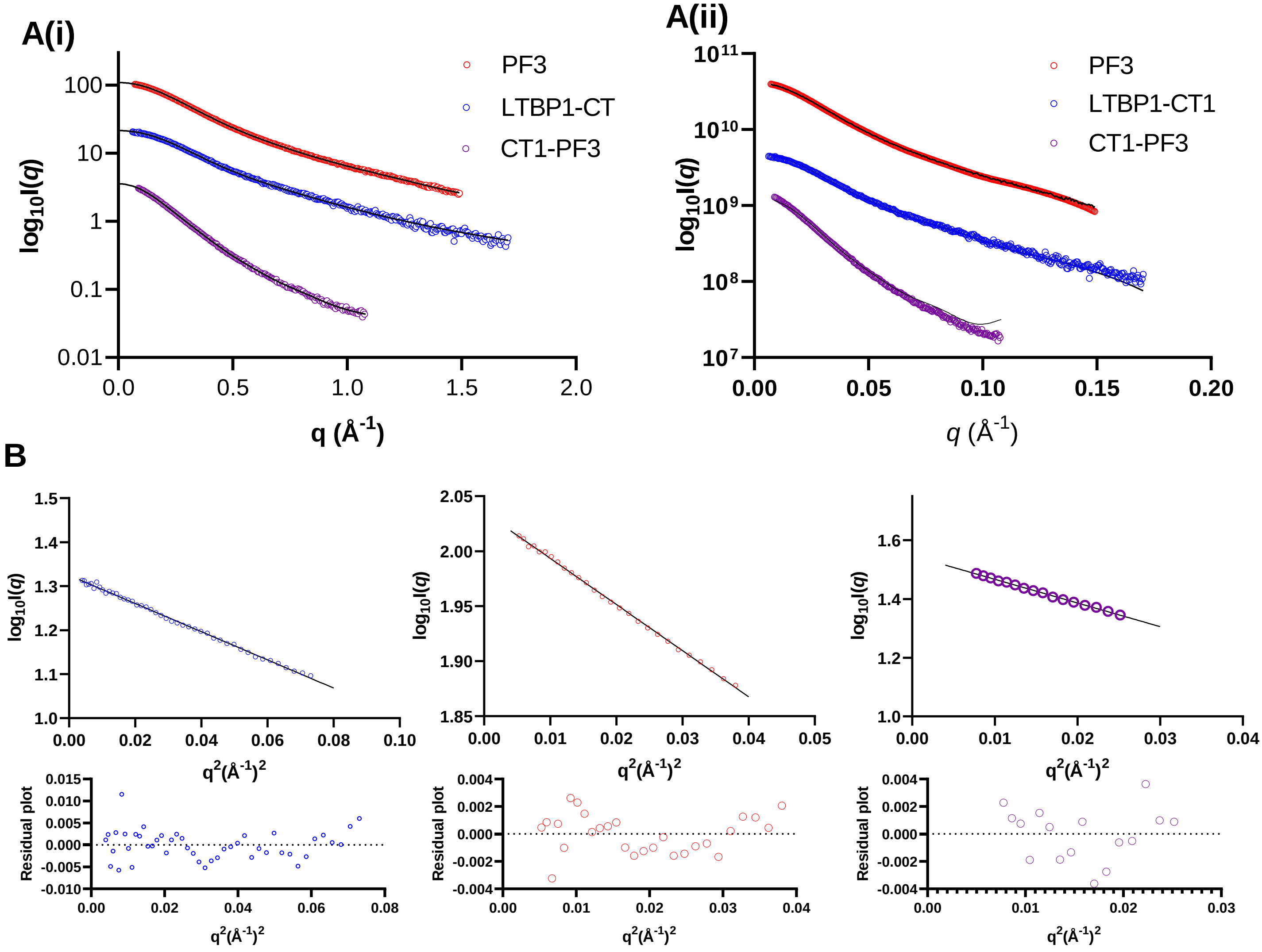

(A) The experimental X-ray scattering intensity (I) plotted as a function of q for fibrillin PF3 (red), LTBP1-CT (blue) and the CT1-PF3 cross-linked complex (purple). In (i) the black lines show the fit of the probability distribution (Pr), shown in figure 5B, to the experimental data and in (ii) the black lines show a representative fit from the multiphase modelling performed using MONSA shown in figure 5C.

(B) Guinier plot with residuals of the low q region for fibrillin PF3 (red), LTBP1-CT (blue) and the CT1-PF3 cross-linked complex (purple) showing linearity and therefore homogeneous samples.

SAXS Data
Supplementary Table 1

	Fibrillin1 PF3	LTBP1 CT	Complex
**Protein Properties**			
Organism	Homo sapiens		
Uniprot sequence ID (residues)	P35555 (45-722 + LVPRGS HHHHHH)	Q14766 (1002–1395 + LVPRGS HHHHHH)	N/A
Mass (Da)	74307.63 (+ 1 glycan)	45592.71 (+ 1 glycan)	119900.34 (+ 2 glycans)
Extinction coefficient [A_{280}, 0.1%(w/v)]	0.757	1.103	0.895
**Data Collection Parameters**			
Source	European Synchrotron Radiation Facility		
Beamline	BM29		
Wavelength (Å)	0.992		
Camera Length (mm)	2.9 m		
q measurement range ($Å^{-1}$)	$0.003 < q < 0.49$		
Detector	Pilatus 1M		
Monitoring Radiation Damage	Frame by frame comparison		
Method of collection	SEC-SAXS (S200 increase 3.2/300)		
Exposure [frame × time(s)]	2000 x 1		
Buffer used	TBS		

SAXS Data
Supplementary Table 2

**Software employed for SAXS data reduction, analysis and interpretation.**	**Fibrillin1 PF3**	**LTBP1 CT**	**Complex**
SAXS data reduction	In-house custom software		
Protein Parameter estimates	ProtParam		
Basic Analysis	Primus		
Bead modelling	MONSA		
Graphical representation	UCSF Chimera – 1.13		

SAXS Data
Supplementary Table 3

	Fibrillin1 PF3	LTBP1 CT	Complex
**Guinier Analysis**			
I(0) (cm^{-1})	109.4	21.53	35.64
R_g (Å)	56.41	50.46	77.88
**P(r) analysis**			
I(0) (cm^{-1})	109.4	21.53	35.64
R_g (Å)	56.70	50.58	78.40
D_{max} (Å)	240	205	294
q_{range} ($Å^{-1}$)	0.072 – 0.149	0.063 – 0.170	0.088 – 0.108
χ^2 (from datgnom)	0.63	0.93	0.63
**Mass estimation (kDa)**			
Baysian	85650	58150	318450
V_c	70428	52031	294553

Supplementary Figure 5

A

```
                                                 *
LTBP1     DLCWEHLSDEYVCSRPLVGKQTTYTECCCLYGEAWGMQCALCPLKDSDDYAQLC
LTBP2     DICWKKVTND-VCSEPLRGHRTTYTECCCQDGEAWSQQCALCPPRSSEVYAQLC
LTBP3     DVCWSQRGEDGMCAGPLAGPALTFDDCCCRQGRGWGAQCRPCPPRGA---GSHC
LTBP4     GVCWQEVGADLVCSHPRLDRQATYTECCCLYGEAWGMDCALCPAQDSDDFEALC
```

B

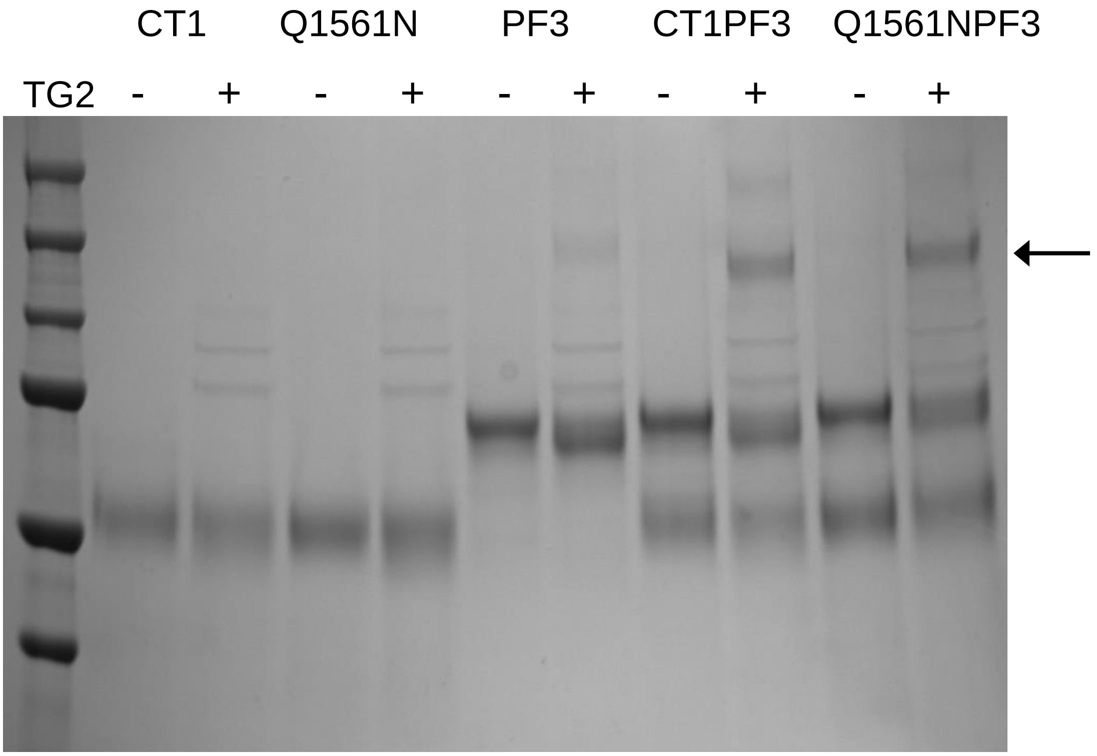

(A) Sequence alignment for the TB3 domain from human LTBP1-4 with Glutamine and Lysine residues highlighted in yellow and cyan, respectively. Residue Q1561 is conserved in LTBP1, 2 and 3 but not LTBP4 and is highlighted by an asterisk.

(B) SDS-PAGE gel stained with Coomassie Blue, showing the fibrillin PF3 fragment and the C-terminal region of LTBP1 (CT1) and a Q1561N mutation in the presence (+) and absence (-) of transglutaminase-2 (TG2). When incubated, complexes form between PF3-CT1 and PF3-CT1-Q1561N shown by an arrow.
